# Ruptured aortic aneurysm due to *Mycobacterium bovis* BCG with a delayed bacteriological diagnosis due to false negative result of the MPB 64 immunochromatographic assay

**DOI:** 10.1186/s13104-017-2382-6

**Published:** 2017-01-26

**Authors:** J. Simar, L. Belkhir, B. Tombal, E. André

**Affiliations:** 1 Microbiology Unit, Laboratory Department, Cliniques Universitaires Saint-Luc, Brussels, Belgium; 2 Infectious Diseases Unit, Department of Internal Medicine, Cliniques Universitaires Saint Luc, Brussels, Belgium; 3 Urology Unit, Department of Surgery, Cliniques Universitaires Saint Luc, Brussels, Belgium; 4 Pôle de Microbiologie Médicale, Institut de Recherche Expérimentale et Clinique, Université Catholique de Louvain, Brussels, Belgium

**Keywords:** *Mycobacterium tuberculosis* complex infections, Case report, BCG therapy, MPB64 immunchromatographic assays

## Abstract

**Background:**

Adjuvant therapy with bacillus Calmette–Guerin (BCG), a live attenuated strain of *Mycobacterium bovis*, has become the treatment of choice for low-risk superficial bladder carcinoma following transurethral resection of the bladder. Complications following vesical BCG instillations are uncommon but, in some cases, severe side-effects can occur such as sepsis or mycotic aneurysm. Besides usual laboratory techniques used for the diagnosis of *Mycobacterium tuberculosis complex* (MTBC) infections (smear microscopy and cultures), commercial immunochromatographic assays detecting MBP64, a 24 kDa *M. tuberculosis* complex-specific secretory protein, can rapidly distinguish MTBC and non-tuberculosis mycobacteria (NTM). MPB64 is found in *M. tuberculosis*, *M. bovis* and some but not all substrains of *M.bovis* BCG. Therefore, these immunochromatographic tests can lead to false negative results and delayed bacteriological diagnosis depending on the presence or absence of MPB64 protein in BCG substrains used for intravesical therapy.

**Case presentation:**

We report the case of a 78-year-old male patient who was admitted to the hospital because of a 1-month history of unexplained fever, thrill, weight-loss and general malaise. His past medical history was marked by a non-muscle-invasive bladder carcinoma treated by transurethral resection followed by BCG instillations (Oncotice, Merck, USA). The patient was initially treated for a urinary tract infection but as fever persists after 72 h of antibiotherapy, urinary tract ultrasound was performed and revealed a large abdominal aortic aneurysm confirmed by computed tomography. Surgery was performed after multidisciplinary discussion. Direct smear of perioperative samples revealed acid-fast bacilli and both solid and liquid cultures were massively positive. Rapid identification of the positive mycobacterial culture was performed using an immunochromatographic assay based on the detection of the *Mycobacterium tuberculosis* MPB 64 antigen. The result was negative for *Mycobacterium tuberculosis* complex. After review of the medical record, a polymerase chain reaction (PCR) was performed and gave a positive result for *M. tuberculosis* complex. Anti-tuberculosis therapy was started immediately and the patient evolved favorably.

**Conclusions:**

Through this case, we showed how the utilisation of MPB64 immunochromatographic assays can provide misleading information due to the variable presence of this protein among the different BCG strains. This case further illustrates the utility of rapid TB complex-specific PCR assays which provide a more reliable identification of all MTBC species.

## Background

The treatment of choice for low-risk superficial bladder carcinoma consists of transurethral resection of the bladder (TURB) followed by intravesical chemotherapy. For high-risk tumours, adjuvant therapy with BCG has become the treatment of choice as it prevents the recurrence and delays disease progression [1]. Mechanism of action of BCG therapy in this particular indication is not fully understood but it seems that the local inflammation induced by these instillations could result in anti-tumor effect [2].

Bacillus Calmette–Guerin (BCG) is a live attenuated strain of *Mycobacterium bovis*, which is part of the *M. tuberculosis* complex (MTBC) and principally used as a vaccine against human tuberculosis [3]. Since the original strain of BCG was characterised in 1921, cumulative genetic mutations have progressively appeared, following decades of sub-culture and global distribution of the strain. This evolution has led to marked differences in the phenotype, the antigenicity (including the secretion of MBP64 protein) and the clinical characteristics between the different vaccinal substrains distributed globally. The MBP64 gene is still present among the substrains Tokyo, Sweden, Moreau, and Russia, but is missing among the substrains Copenhagen, Glaxo, Pasteur and Tice (the strain used for the treatment of the patient presented below) [4–6].

Complications following BCG instillations or vaccinations are uncommon (1–10% of patients) and usually consist in local reactions (including hypersensitivity reactions, abscesses at the injections site and localized lymphadenopathy). Severe disseminated infections following vaccination have been reported, but this complication is infrequent and usually linked to severe alteration of the immunity [7]. On the other hand, over one-third of patients report moderate general symptoms (fever, malaise), hematuria, pollakiuria or dysuria following BCG bladder instillations. 1–4% of patients further present granulomatous prostatitis or epididymo-orchitis. Life-threatening BCG sepsis is uncommon (<1% of patients). Mycotic aneurysm is an extremely rare complication, involving predominantly the aorta. To date, less than 30 patients with mycotic aneurysm following intravesical BCG therapy have been reported. Globally, local and systemic side-effects result in treatment discontinuation for approximately 20% of patients [8, 9].

In order to prevent systemic complications, BCG instillations are contraindicated during a period of 2 weeks following TURB, bladder biopsy, traumatic catheterization, urethral stenosis, macroscopic haematuria, prior BCG sepsis and urinary tract infection. It is also contraindicated in the context of active tuberculosis infection [10].

In most clinical laboratories, the bacteriological diagnosis of *M. bovis* BCG infections is performed using the same laboratory techniques as for MTBC infections, including smear microscopy and culture. Among positive cultures, the differentiation between MTBC and NTM is done using commercial immunochromatographic assays detecting the MBP64 antigen in positive cultures [11]. In Belgium, direct MTBC PCR is currently not recommended outside particular indications.

We report the case of a patient with a ruptured abdominal mycotic aortic aneurysm following intravesical BCG instillation for whom the bacteriological diagnostic was delayed due to a false negative result of the BD MGIT TBc Identification Test©.

## Case presentation

A 78-year-old male patient was admitted to the hospital following a 1-month history of unexplained fever, thrill, weight-loss and general malaise, and a 1-week complaint of pollakiuria.

Twelve years prior to this episode, the patient was diagnosed with a non-muscle-invasive bladder carcinoma that was treated by transurethral resection followed by BCG instillations (Oncotice, Merck, USA). These instillations were repeated after 7, 8 and 11 years due to oncological relapse. The last BCG instillation was administered 5 months before admission.

The medical history of the patient further included hypertension, and atrial fibrillation for which he received anti-vitamin K therapy. He was in remission of a prostate cancer for which he had received radiotherapy and anti-androgen therapy 4 years earlier.

On admission, clinical examination was unremarkable. Laboratory workup revealed inflammation (CRP elevated at 5.7 mg/dL), hematuria and leucocyturia. Urine culture was positive for *Escherichia coli* and cefuroxime antibiotherapy was initiated according to the drug susceptibility profile.

**Fig. 1:**
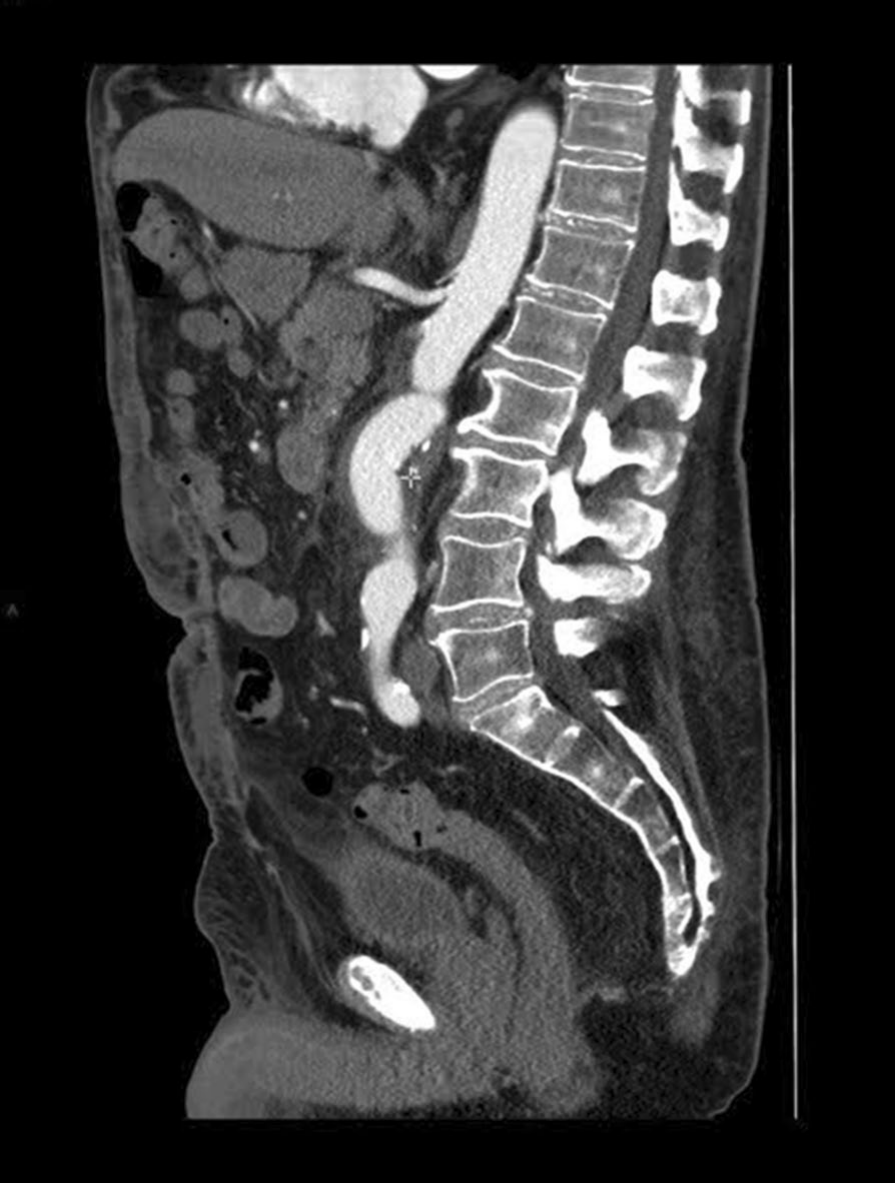
CT sagittal section performed showing abdominal aortic aneurysm. A CT was performed after initial failure of antibiotherapy and showed and abdominal aortic aneurysm previously seen on US

As fever was persisting 72 h after initiation of antibiotic therapy, a urinary tract ultrasound (US) was performed in order to exclude an obstacle or an abcess. US revealed a large abdominal aortic aneurysm, which was subsequently confirmed by computed tomography (CT). The size of the aneurysm was measured at 7 × 7 × 7.3 cm and located in the infra-renal region (Fig. 1). This image was not present on a CT performed 3 years earlier. Antibiotic therapy was stopped and blood cultures were collected.

Four days after admission, the patient fell during the night. A second abdominal CT revealed a large retroperitoneal hematoma but no sign of rupture was observed. After multi-disciplinary discussion, the patient underwent surgery. Perioperative samples were sent to the bacteriology laboratory for conventional and mycobacterial cultures.

Direct smear of the aneurysm was positive for acid-fast bacilli. Direct PCR was not performed, as current Belgian guidelines do not recommend to perform this test on non-pulmonary samples. Both liquid cultures (MGIT, Becton–Dickinson, Baltimore, MD, USA) and solid cultures (Löwenstein-Jensen media, Beckton Dickison, Baltimore, MD, USA) were positive. Following internal procedures and the manufacturer’s instructions, rapid identification of the positive mycobacterial culture was performed using the BD MGIT TBc Identification Test© (Beckton Dickinson Diagnostic, USA), an immunochromatographic assay based on the detection of the *Mycobacterium tuberculosis* MPB 64 antigen. The result was negative for *Mycobacterium tuberculosis* complex. After review of the medical record, BCGosis was suspected, and the PCR-based Xpert MTB/Rif assay (Cepheid, USA) was performed on the positive culture and gave a positive result for *M. tuberculosis* complex. *M.bovis* BCG identification was later confirmed by the Belgian national reference center which performed a second PCR targeting CSB and RD1 [12].

Management of *M. bovis* mycotic aneurysms include both surgical replacement with an aortic graft material and 9–12 months antimycobacterial therapy including isoniazid, rifampicine and ethambutol [6]. Pyrazinamide is not used due to intrinsic resistance of *M. bovis* to this drug. Antimycobacterial therapy decreases the risk of relapse following surgery [13].

As a result, anti-tuberculosis therapy with rifampicine, ethambutol, isoniazide and pyrazinamide was started in our patient. Pyrazinamide was discontinued upon definitive identification of BCG. After 2 months, ethambutol was discontinued and rifampicin and isoniazide were pursued for additional 7 months. The patient showed no signs of relapse after two-years of follow up. The clinical examination and the CT were normal at that time.

## Conclusion

Mycotic aneurysm is an extremely rare but life-threatening complication of intravesical BCG therapy, generally involving the aorta [14]. Rapid and accurate diagnosis is important to ensure the prompt initiation of the adequate treatment. Through this case, we showed how the utilisation of MPB64 immunochromatographic assays can provide misleading information due to the variable presence of this protein among the different BCG strains. This case further illustrates the superiority of rapid TB complex-specific PCR assays which provide a more reliable identification of all MTBC species. Following this experience, the laboratory has modified its internal procedures, and a PCR is now systematically performed for all samples presenting a direct smear or a positive culture. In the latter case, the MPB64 immunochromatographic assay is still performed in parallel.

MPB64 immunochromatographic still remains, in most cases, a useful tool for the differential diagnosis between MTBC and NTM infections. The present cases nevertheless suggests that this simple assay should be replaced by PCR assays for patients presenting a risk of BCG-related infection.

## Abbreviations

BCG: bacillus Calmette–Guerin
MTBC: *Mycobacterium Tuberculosis* complex
NTM: non-tuberculosis mycobacteria
PCR: polymerase chain reaction
TURB: transurethral resection of the bladder
US: ultrasound
CT: computed tomography
